# Supplementary figures and images for: Reanalysis of single-cell RNA sequencing data does not support herpes simplex virus 1 latency in non-neuronal ganglionic cells in mice
Source: bioRxiv. 2023 Jul 18:2023.07.17.549345. Preprint. [Version 1] doi: 10.1101/2023.07.17.549345 (PMC10370134; doi:10.1101/2023.07.17.549345)

## B cell

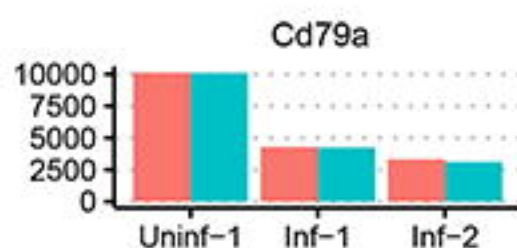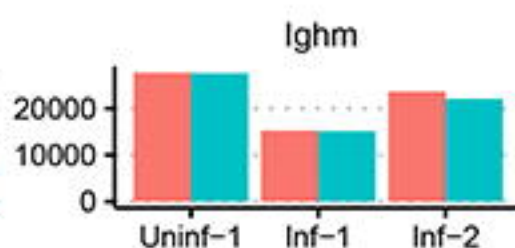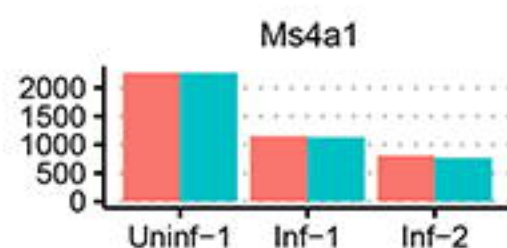

## endothelial cell

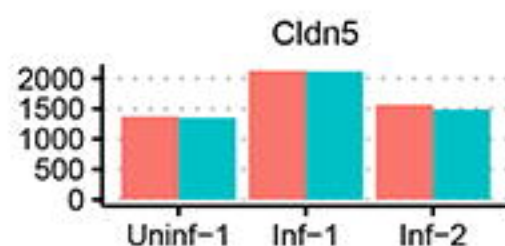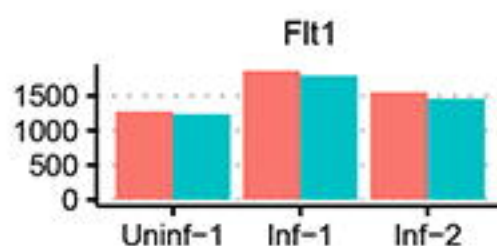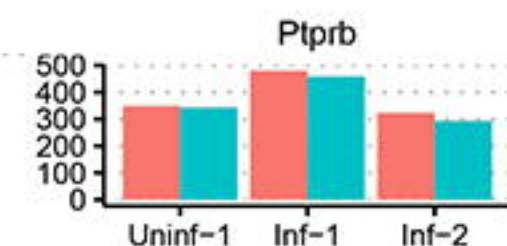

## neuron

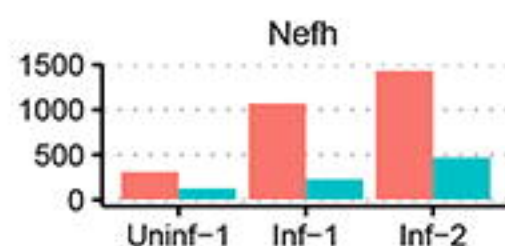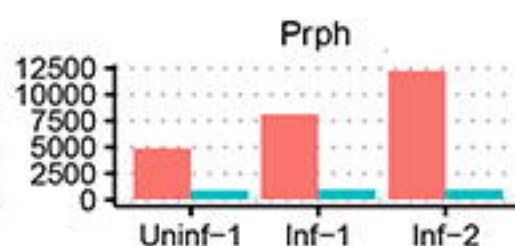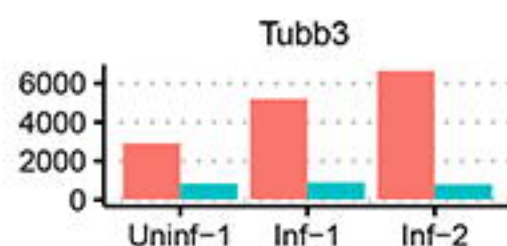

## Neutrophils

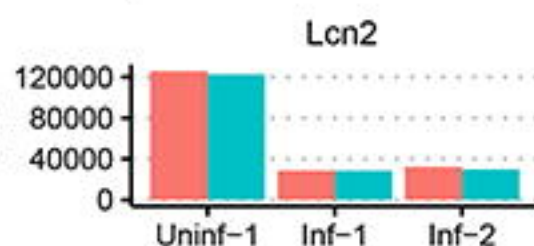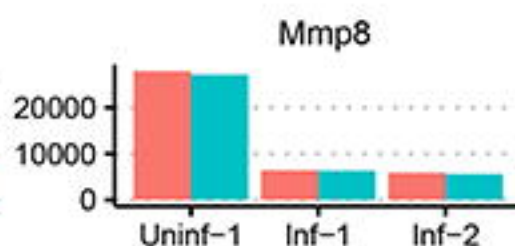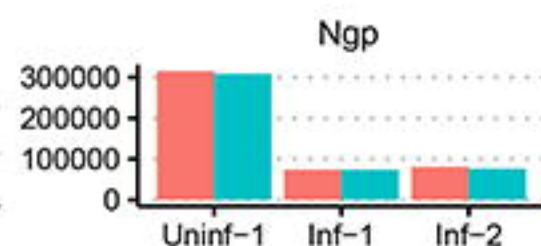

## Schwann cell

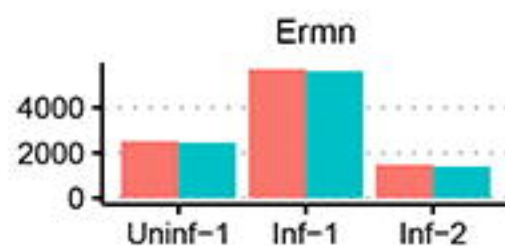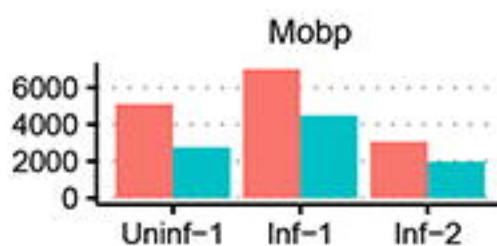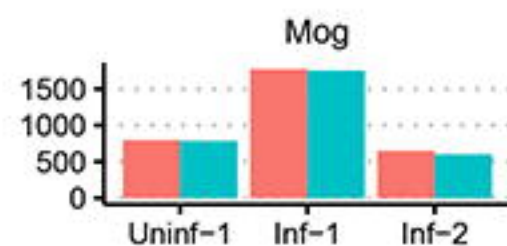

## T cell

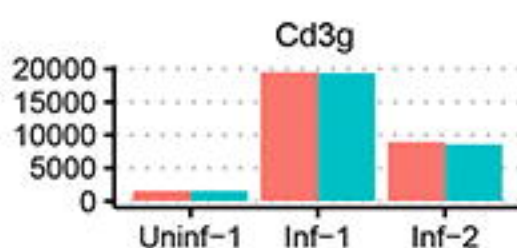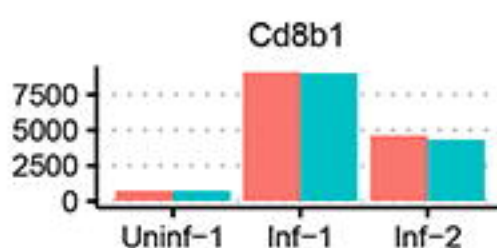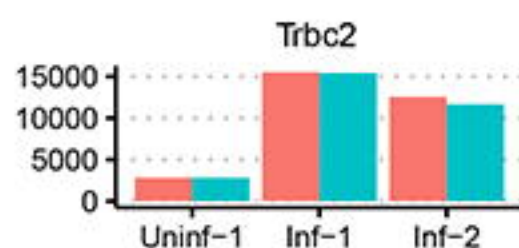

## HSV-1

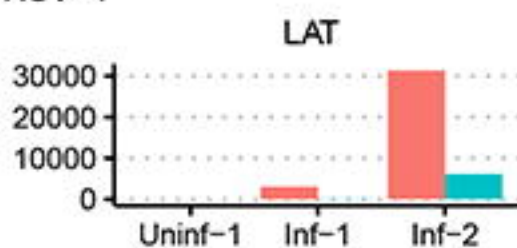

pre-filter  
post-filter

Supplement: 1 — Figure S1. Further evidence that abundant loss of reads during filtering is specifically associated with HSV-1 LAT and neuronal markers. For HSV-1 LAT and representative markers of different cell types, we determined the total number of reads present in the pre-filtered and post-filtered datasets. [file NIHPP2023.07.17.549345V1-supplement-1.pdf]
